# Clinical outcome and prognostic factors in elderly traumatic brain injury patients receiving neurointensive care

**DOI:** 10.1007/s00701-019-03893-6

**Published:** 2019-04-13

**Authors:** Samuel Lenell, Lena Nyholm, Anders Lewén, Per Enblad

**Affiliations:** Department of Neuroscience/Neurosurgery, Section of Neurosurgery, Uppsala University Hospital, Uppsala University, SE-751 85 Uppsala, Sweden

**Keywords:** Traumatic brain injury, Elderly, Outcome, Quality register, Neurointensive care, Prognostic factors

## Abstract

**Background:**

The probability of favorable outcome after traumatic brain injury (TBI) decreases with age. Elderly, ≥ 60 years, are an increasing part of our population. Recent studies have shown an increase of favorable outcome in elderly over time. However, the optimal patient selection and neurointensive care (NIC) treatments may differ in the elderly and the young. The aims of this study were to examine outcome in a larger group of elderly TBI patients receiving NIC and to identify demographic and treatment related prognostic factors.

**Methods:**

Patients with TBI ≥ 60 years receiving NIC at our department between 2008 and 2014 were included. Demographics, co-morbidity, admission characteristics, and type of treatments were collected. Clinical outcome at around 6 months was assessed. Potential prognostic factors were included in univariate and multivariate regression analysis with favorable outcome as dependent variable.

**Results:**

Two hundred twenty patients with mean age 70 years (median 69; range 60–87) were studied. Overall, favorable outcome was 46% (Extended Glasgow Outcome Scale (GOSE) 5–8), unfavorable outcome 27% (GOSE 2–4), and mortality 27% (GOSE 1). Significant independent negative prognostic variables were high age (*p* < 0.05), multiple injuries (*p* < 0.05), GCS M ≤ 3 on admission (*p* < 0.05), and mechanical ventilation (*p* < 0.001).

**Conclusions:**

Overall, the elderly TBI patients > 60 years receiving modern NIC in this study had a fair chance of favorable outcome without large risks for severe deficits and vegetative state, also in patients over 75 years of age. High age, multiple injuries, GCS M ≤ 3 on admission, and mechanical ventilation proved to be independent negative prognostic factors. The results underline that a selected group of elderly with TBI should have access to NIC.

## Introduction

Outcome after traumatic brain injury (TBI) has improved over time with the development of neurointensive care (NIC) [3, 4, 9, 10, 26, 27, 30, 48] despite the fact that favorable outcome decreases with increasing age [16, 21, 28, 29, 38, 44], and there is an increasing proportion of elderly in the TBI population [17, 19, 33, 34]. The United Nations reports that the population aged 60 or older is growing faster than all the younger age groups and expects the number of persons over 60 years to be more than doubled by 2050 [46]. Elderly are prone to trauma from falls. One third of every person above 60 years and every other person above 80 years have a falling accident every year [23]. The management of elderly patients with traumatic head injury constitutes a tremendous challenge in the future. An updated periodic evaluation of NIC of TBI patients made by us showed substantial increase of the proportion of patients > 60 years treated from 16 to 30% between 1996–1997 and 2008–2009 [22]. Furthermore, when clinical outcome was evaluated in the elderly TBI patients who received NIC, 51% of patients age ≥ 65 had favorable outcome [28]. Those relatively favorable results indicate that elderly patients with TBI should not be excluded from NIC. However, the optimal patient selection and most beneficial treatments may differ in the elderly and the young. Elderly patients have comorbidities to a higher degree, are more likely to use anticoagulants, and respond less well to rehabilitation [5]. Therefore, it is important to gain more knowledge about elderly TBI patients. The aims of this study were to examine outcome in a larger group of elderly TBI patients receiving NIC and to identify demographic- and treatment-related prognostic factors specifically in the elderly.

## Material and methods

### Referral of patients

The Department of Neurosurgery at the Uppsala University Hospital in Sweden provides highly specialized NIC for a population of approximately 2 million people living in the central part of Sweden. Patients arriving at local hospitals are stabilized according to the ATLS principles and then referred to Uppsala for tertiary care (the most distant local hospital 382 km away) [11].

### Patient selection and data collection

Information about clinical characteristics, management, and clinical outcome are recorded for all TBI patients treated at the NIC unit in Uppsala in the Uppsala Traumatic Brain Injury register [31].TBI patients ≥ 60 years of age registered between 2008 and 2014 were eligible for the study. In total, 249 patients were identified. After exclusion of 29 elderly patients, 220 remained to be the studied. The patients were excluded for the following reason: patients admitted to the NIC unit ≥ 5 days after the trauma (*n* = 10), or treated successfully at the NIC unit within 24 h (*n* = 6); patients with both pupils wide and non-reacting on arrival at the NIC unit (*n* = 4) (i.e., patients with an obvious predestined fatal clinical course [1, 7]); patients with gunshot wound to the head (*n* = 1); patients lost to follow-up (*n* = 8).

### Data studied

The following parameters were studied: primary or secondary transfer, sex, age, cause of trauma, multiple injuries, trauma under influence of drugs/alcohol, acute surgery before arrival, GCS on admission, medical history (brain injury/disease, previous traumatic brain injury, diabetes mellitus, hypertension/cardiovascular disease (CVD), antithrombotic drugs (subgrouped by antiplatelet, warfarin, non-vitamin K antagonist oral anticoagulants (NOAC), and low molecular weight heparin (LMWH)), and ethylism), craniotomy, cause of craniotomy, decompressive hemicraniectomy, intracranial pressure (ICP) monitoring, mechanical ventilation, and NIC mortality.

### Radiology

The computed tomography (CT) scans from the admission were classified retrospectively according to Marshall Classification [25] by one of the authors (S.L.).

### Neurointensive care

All patients were treated according to the standardized escalated management protocol, described in detail earlier [10], and summarized below:

#### Basal treatment

All unconscious patients (Glasgow Coma Scale motor response (GCS M) ≤ 5) are intubated and mechanically ventilated. Intubated patients are moderately hyperventilated (PaCO_2_ 4.0–4.5 kPa) on admission with the aim of normoventilation as soon as possible when ICP allows. Propofol (Propofol-LipuroB; Braun Medical, Danderyd, Sweden) is used for sedation and morphine for analgesia. ICP is monitored in unconscious patients using an external ventricular drain (EVD) or an intraparenchymal pressure probe. When EVD is used, ICP is measured with the pressure dome at the level of the lateral ventricles. Arterial blood pressure is measured with the pressure dome at heart level. Patients are positioned in bed with 30° head elevation to facilitate venous outflow. Clinical neurological status is monitored using frequent wake-up tests. Lesions causing significant mass effect, extracerebral hematomas or contusions, are surgically evacuated except when coagulopathy is resistant to therapy. Prophylactic anticonvulsants are not used. Thromboprophylaxis are used when the risk for new intracranial bleedings are deemed low and continued until patients have been mobilized. Treatment goals are as follows: ICP < 20 mmHg, cerebral perfusion pressure (CPP) > 60 mmHg, systolic blood pressure (SBP) > 100 mmHg, central venous pressure (CVP) 0–5 cm H_2_0, pO_2_ > 12 kPa, blood glucose 5–10 mmol/L, electrolytes within normal range, normovolemia, and body temperature < 38 °C. If ICP is increased > 20 mmHg without mass lesions, intermittent cerebrospinal fluid (CSF) drainage of small volumes (1–2 ml) are used during the early period when there are risks of expanding hematomas and brain swelling. Later, CSF is drained using an open system against a pressure level of 15–20 mmHg if needed.

#### Step 1A

In case of persisting ICP problems, the treatment is escalated to Step 1A with no wake-up test. This entails continuous sedation with propofol and stress reduction with β1-antagonist metoprolol (Seloken®, AstraZeneca AB Södertälje, Sweden) (0.2–0.3 mg/kg/24 h as an infusion) and α2-agonist clonidin (Catapresan®, BoehingerIngelheim AB Stockholm Sweden) (0.5–1.0 μg/kg × 8 or the same dose as an infusion).

#### Step 1B

When the ICP problems continue, barbiturate coma treatment with infusion of thiopental (Pentocur, Abcur AB, Helsingborg, Sweden) is initiated provided that there is no shift of the midline. Bolus dose of 4–8 mg/kg is given as repeated 50 mg injections until ICP is < 20 mmHg followed by an infusion of 5–10 mg/kg/h for 6 h and thereafter 2–5 mg/kg/h as required to control ICP. The lowest possible dose is used to keep ICP < 20 mmHg and burst-suppression on electroencephalogram (EEG) is not the goal. During this treatment, a CPP as low as 50 mmHg is allowed. Thiopental concentration > 380 μmol/L is avoided. Because of the high risk of severe side effects with barbiturate coma treatment in elderly, this therapy was only exceptionally escalated to this step in old patients.

#### Step 2

Decompressive craniectomy [42] is used when Step 1B is insufficient to reduce ICP or when adverse effects of the thiopental treatment are observed. Bi-fronto-temporal craniectomies are done, sparing the bone ridge in the midline when there are no mass lesions. When there is a shift of the midline and no localized mass lesions to evacuate, a hemicraniectomy is done.

### Evaluation of outcome

Clinical outcome was assessed after around 6 months using structured telephone interviews for the Extended Glasgow Outcome Scale (GOSE) [39, 43]. The interview was done by a few selected persons.

The outcome was categorized in favorable (GOSE 5–8), unfavorable (GOSE 2–4), and dead (GOSE 1).

### Statistical methods

To compare different age groups, Pearson’s Chi-squared test was used. Patients and treatment factors were analyzed using univariate logistic regression. Multivariate logistic regression analysis was performed with favorable outcome (GOSE 5–8) as dependent variable. Admission variables were included as explanatory variables, and admission together with treatment variables was also analyzed. All explanatory variables were dichotomized except age. IBM SPSS Statistics for Windows was used.

## Results

### Age distribution

**Fig. 1:**
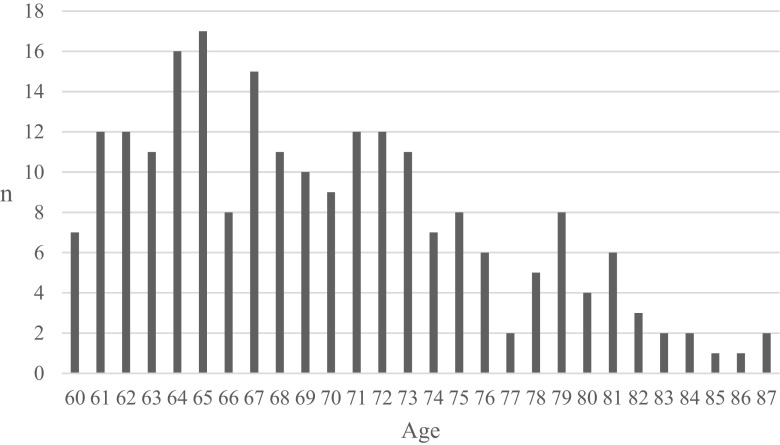
Age distribution

The mean age of the 220 patients was 70 years (median 69; range 60–87). The age distribution showed that most of the patients were between 60 and 75 years (Fig. 1).

### Patient characteristics on admission

The patient characteristics are presented in Table 1 for all patients > 60 years old, for patients 60–74 years old, and for patients 75–89 years old. There were 170 patients 60–74 years old and 50 patients 75–89 years old. There was no significant difference in sex between the two age groups. The most common cause of trauma was falls which occurred in 77% of all cases (170 patients). There was no significant difference between the two age groups. In both age groups, around 90% of the patients were admitted in GCS M ≥ 4. Multiple injuries were found in 25% of the 60–74-year-old patients and in 10% of the 75–89 years old (*p* < 0.05). Trauma under the influence of alcohol was almost 5 times as common in the 60–74-year-old patients compared to the older patients, 26% vs 6%, respectively, (*p* < 0.01).

**Table 1:** Characteristics on admission

Patient and trauma characteristics	All		60–74		75–89		*p* 60–74 vs 75–89	
	*n*	%	*n*	%	*n*	%		
Total	220		170		50			
Referrals	167	76	140	82	27	54	0.000	***
Sex (female)	61	28	44	26	17	34	0.260	
Male	159	72	126	74	33	66	0.260	
Multiple injuries	47	21	42	25	5	10	0.026	*
Under the influence of drugs/alcohol at trauma	47	21	44	26	3	6	0.003	**
Cause of trauma								
Bicycle accident	7	3	6	4	1	2		
Fall accident	170	77	132	78	38	76	0.807	
Vehicle accident	20	9	16	9	4	8		
Pedestrian hit by vehicle	9	4	6	4	3	6		
Assault	3	31	3	2	0	0		
Sports injury	1	0	0	0	1	2		
Other	10	5	7	4	3	6		
GCS motor response								
6 Obeys commands	106	48	80	47	26	52	0.539	
5 Localizes pain	68	31	55	32	13	26	0.393	
4 Withdraws (normal flexion)	24	10	17	1	7	14	0.425	
3 Stereotyped flexion	11	5	9	6	2	4		
2 Stereotyped extension	6	3	4	2	2	4		
1 None	5	2	5	3	0	0		
GCS M ≥ 4 on admission	198	90	152	89	46	92	0.592	
GCS M ≤ 3 on admission	22	10	18	11	4	8	0.592	
Dominating injury type on CT								
ASDH	95	43	57	34	38	76	0.000	***
Other	3	1	2	1	1	2		
DAI	2	1	2	1	0	0		
EDH	4	2	4	2	0	0		
Impression fracture^a^	3	1	3	2	0	0		
Contusions	64	29	59	35	5	10	0.001	***
Mixed	26	12	23	14	3	6	0.147	
Normal CT	0	0	0	0	0	0		
Traumatic SAH	23	10	20	12	3	6	0.242	
Initial CT Marshall Classification								
Diffuse injury I	2	1	2	1	0	0		
Diffuse injury II	80	36	69	41	11	22	0.016	*
Diffuse injury III	21	9	19	11	2	4		
Diffuse injury IV	14	6	9	5	5	1		
Evacuated mass lesion	68	31	48	28	20	40	0.114	
Non-evacuated mass lesion	35	16	23	14	12	24	0.075	
Diffuse injury I–IV	117	53	99	58	18	36	0.006	**
Focal mass lesion	103	47	71	41	32	64	0.006	**
Medical history of								
Brain injury/disease	45	20	33	19	12	24	0.480	
Traumatic brain injury	8	4	7	4	1	2		
Diabetes mellitus	36	16	25	15	11	22		
Hypertension/CVD	118	54	82	48	36	72	0.003	**
Ethylism	56	25	51	30	5	10	0.004	**
Antithrombotic drugs	82	37	51	30	31	62	0.000	***
Antiplatelet	48	22	36	16	12	24	0.671	
Warfarin	34	15	13	8	21	42	0.000	***
NOAC	8	4	7	4	1	2		
LMWH	6	3	4	2	2	4		

^a^All impression fractures also hade intracerebral or subarachnoidal blood

*p < 0.05

**p < 0.01

***p < 0.001

Overall, the most common type of injury dominating TBI was acute subdural hematoma (ASDH; 43%) followed by contusions (29%). In the 60–74-year-old group, contusions were the dominating injury type, and occurred in 35% of the patients, compared to 10% in the 75–89-year-old group (*p* < 0.001). In the 75–89-year-old group, the dominating injury type was ASDH, occurring in 76% of the patients compared to 34% in the 60–74 years old (*p* < 0.01) (Table 1).

When the initial CT scans were classified according to Marshall Classification (Table 1), diffuse injury II was the most common class with 41% in patients 60–74 years old and 22% in patients 75–89 years old (*p* < 0.05). Evacuated mass lesion was the most common Marshall Classification in patients 75–89 years old and occurred in 40% of those patients.

Regarding the medical history (Table 1), 54% of all elderly had hypertension/CVD and 37% used antithrombotic drugs. One fifth of all 220 patients (20%) had a history of previous brain injury/disease before the trauma, but only 4% of those were a previous TBI. Among patients 60–74 years old, 48% had hypertension/CVD in the medical history compared to 72% in patients 75–89 years old (*p* < 0.01). Antithrombotic drugs were almost twice as common in 75–89-year-old patients compared to 60–74-year-old patients, 62% and 30%, respectively (*p* < 0.001). Looking at the specific antithrombotic drugs, warfarin was four times as common in 75–89-year-old patients compared to patients 60–74 years old; 42% vs 8%. Among patients 60–74 years old, 30% of had a history of ethylism compared to 10% among patients 75–89 years old (*p* < 0.01).

### Management characteristics

Among all 220 elderly patients, 177 (80%) received mechanical ventilation for a mean of 7 days (median 6, range 1–21), and 118 (53%) had ICP monitoring for a mean of 10 days (median 8, range 2–25) (Table 2). Eighteen patients (8%) had been operated with evacuation of ASDH at the referring hospital due to acute herniation before arrival (Table 2). Ninety-five patients (43%) had a craniotomy done during NIC, most commonly due to ASDH which occurred in 80 patients (36%) followed by evacuation of contusions in 25 patients (11%). Decompressive hemicraniectomy was done in 9 patients (4%). Thirty patients 75–89 years old (60%) had a craniotomy compared to 65 patients 60–74 years old (38%) (*p* ≤ 0.01). Three patients received thiopental.

**Table 2:** Management characteristics

Management	All		60–74		75–89		*p* 60–74 vs 75–89	
	*n*	%	*n*	%	*n*	%		
Total	220		170		50			
Emergency craniotomy before arrival	18	8	13	8	5	10		
Craniotomy	95	43	65	38	30	60	0.006	**
Evacuation extracerebral hematoma^b^	87	40	58	34	29	58	0.002	**
Evacuation EDH	3	1	3	2	0	0		
Evacuation ASDH	80	36	52	31	28	56	0.001	**
Evacuation for both (EDH + SDH)	4	2	3	2	1	2		
Evacuation contusions^b^	25	11	21	12	4	8	0.394	
Decompressive hemicraniectomy	9	4	7	4	2	4		
Multiple surgeries	22	10	14	8	8	16	0.108	
ICP monitoring	118	53	96	56	22	44	0.120	
EVD only	21	10	19	11	2	4		
Intraparenchymal probe only	76	35	56	33	20	40	0.356	
EVD and intraparenchymal probe	21	10	21	12	0	0		
Days with ICP monitoring (mean)	9.5		10		7.4			
Mechanical ventilation	177	80	135	79	42	84	0.472	
Days with mechanical ventilation (mean)	7.4		7.6		6.8			

^b^Some patients evacuated both extracerebral hematoma and contusions

*p < 0.05

**p < 0.01

***p < 0.001

### Clinical outcome

**Fig. 2:**
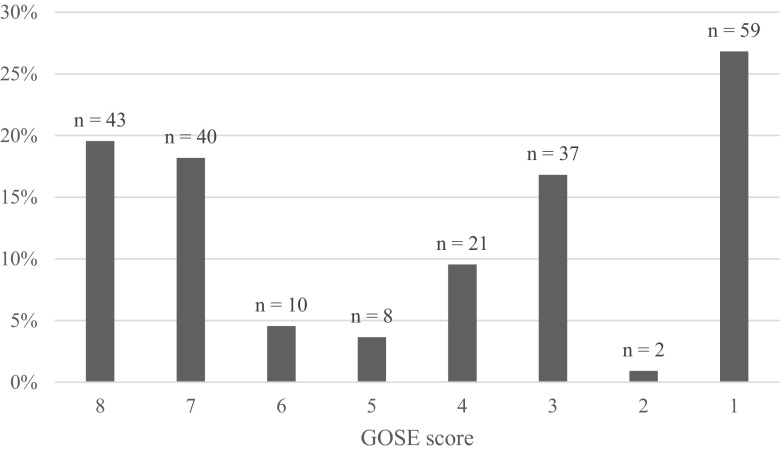
GOSE score on 6-month follow-up

**Fig. 3:**
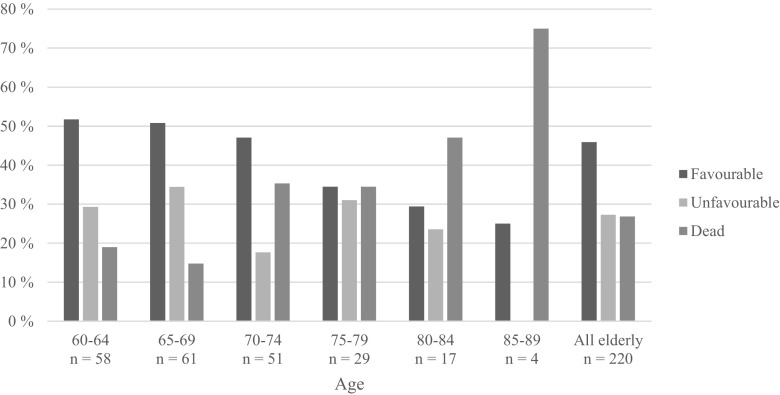
Categorized outcome by age subdivided by 5 years. Outcome categorized in favorable (GOSE 5–8), unfavorable (GOSE 2–4), and dead (GOSE 1)

Follow-up of surviving patients was made after 7.8 months in mean (median 7, range 5–28). When outcome was graded with the Extended Glasgow Outcome Scale, 43 patients (20%) were GOSE 8 (upper good recovery), 40 (18%) were GOSE 7 (lower good recovery), 10 (5%) were GOSE 6 (upper moderate disability), 8 (4%) were GOSE 5 (lower moderate disability), 21 (10%) were GOSE 4 (upper severe disability), 37 (17%) were GOSE 3 (lower severe disability), 2 patients (1%) were in GOSE 2 (vegetative state), and 59 patients (27%) were GOSE 1 (dead; 17 (8%) died at the NICU) (Fig. 2). The clinical outcome by age groups is summarized in Fig. 3. Patients 60–69 years old showed favorable outcome in around 50% of the cases and < 20% died. Patients 70–74 years old almost also showed favorable outcome in 50% of the cases and around 35% died. In patients 75–84 years of age, favorable outcome was around 30% and declined to 25% in patients 85–89 years old. Of the 60–74 years old, 11 patients (6%) died at the NICU compared with 6 (12%) in the 75–89 years old.

### Prediction of prognosis

Univariate logistic regression analysis with favorable outcome (GOSE 5–8) as dependent variable (Table 3) showed the following significant patient variables (predictors): age (*p* < 0.05), GCS M ≤ 3 on admission (*p* < 0.01), diffuse injury Marshall score I–IV (*p* < 0.001), and Marshall score evacuated mass lesion (EML) (*p* < 0.001) and warfarin (*p* < 0.05). The following patient variables showed marginal significance (Table 3): extracerebral hematoma (*p* = 0.08), history of brain injury/disease (*p* = 0.056), and history of ethylism (*p* = 0.066) and antiplatelet (*p* = 0.053).

**Table 3:** Predictive value of admission and treatment variables for favorable outcome (univariate logistic regression analysis with favorable outcome (GOSE 5–8) as dependent variable)

Variables	Odds ratio	95% CI		*p*	
		Lower	Upper		
Age	0.948	0.908	0.989	0.013	*
Sex (female)	1.095	0.606	1.979	0.764	
GCS M ≤ 3 on admission	0.161	0.046	0.562	0.004	**
Multiple injuries	0.753	0.391	1.449	0.396	
Under the influence of drugs/alcohol at trauma	0.728	0.378	1.403	0.343	
Marshall Classification					
Diffuse injury I–IV	3.189	1.828	5.565	0.000	***
EML	0.299	0.160	0.560	0.000	***
NEML	0.751	0.360	1.567	0.445	
CT dominating injury type					
Extracerebral hematoma	0.619	0.361	1.059	0.080	
Contusions	1.646	0.916	2.956	0.096	
All other	1.082	0.591	1.981	0.797	
Medical history of					
Brain injury/disease	0.512	0.257	1.017	0.056	
Traumatic brain injury	0.160	0.019	1.323	0.089	
Diabetes mellitus	0.806	0.391	1.661	0.558	
Hypertension/CVD	0.900	0.528	1.534	0.699	
Ethylism	0.556	0.297	1.040	0.066	
Antithrombotic drugs	1.028	0.594	1.779	0.921	
Antiplatelet	1.899	0.993	3.632	0.053	
Warfarin	0.435	0.197	0.960	0.039	*
NOAC	1.186	0.289	4.866	0.813	
LMWH	1.184	0.234	5.998	0.839	
Surgery before arrival	0.845	0.326	2.188	0.728	
Craniotomy	0.412	0.237	0.716	0.002	**
Evacuated extracerebral hematoma	0.498	0.286	0.868	0.014	*
Evacuated contusions	0.418	0.167	1.045	0.062	
Decompressive hemicraniectomy	0.323	0.066	1.592	0.165	
Multiple surgeries	1.200	0.497	2.897	0.685	
ICP monitoring	0.634	0.371	1.082	0.095	
Mechanical ventilation	0.253	0.122	0.526	0.000	***

*p < 0.05

**p < 0.01

***p < 0.001

For the treatment variables, the significant variables were (Table 3): craniotomy (*p* < 0.01), evacuation of extracerebral hematoma (*p* < 0.05), and mechanical ventilation (*p* < 0.001).

Multivariate logistic regression analysis of admission variables showed that the significant independent variables were age (*p* < 0.05) and multiple injuries (*p* < 0.05). GCS M ≤ 3 on admission (*p* = 0.052) and EML (*p* = 0.078) showed marginal significance (Table 4).

**Table 4:** Prediction model of admission variables for favorable outcome (multivariate logistic regression analysis with favorable outcome (GOSE 5–8) as dependent variable)

Variables	Regression coefficient	SE	Wald *X*^2^	Odds ratio	95% CI		*p*	
					Lower	Upper		
Intercept	4.114	2.032	4.101	61.192			0.043	*
Age	− 0.055	0.028	3.860	0.947	0.897	1.000	0.049	*
Sex (female)	0.056	0.366	0.023	1.057	0.516	2.167	0.879	
GCS M ≤ 3 on admission	− 1.350	0.696	3.765	0.259	0.066	1.014	0.052	
Multiple injuries	− 0.997	0.415	5.779	0.369	0.164	0.832	0.016	*
Under the influence of drugs/alcohol at trauma	− 0.135	0.492	0.076	0.874	0.333	2.290	0.783	
Marshall Classification								
Diffuse injury I–IV	0.521	0.478	1.189	1.684	0.660	4.300	0.275	
EML	− 0.910	0.516	3.114	0.403	0.147	1.106	0.078	
CT dominating injury type								
Extracerebral hematoma	0.464	0.458	1.027	1.591	0.648	3.903	0.311	
Contusions	0.296	0.416	0.508	1.345	0.595	3.036	0.476	
Medical history of								
Brain injury/disease	− 0.514	0.440	1.362	0.598	0.252	1.418	0.243	
Traumatic brain injury	− 1.425	1.259	1.281	0.240	0.020	2.837	0.258	
Diabetes mellitus	− 0.586	0.457	1.648	0.557	0.227	1.362	0.199	
Hypertension/CVD	0.015	0.387	0.001	1.015	0.476	2.165	0.970	
Ethylism	− 0.648	0.490	1.747	0.523	0.200	1.368	0.186	
Antithrombotic drugs								
Antiplatelet	0.540	0.434	1.548	0.523	0.200	1.368	0.213	
Warfarin	− 0.764	0.517	2.184	1.717	0.733	4.021	0.139	
NOAC	− 0.661	0.824	0.644	0.466	0.169	1.283	0.422	
LMWH	− 0.597	1.024	0.340	0.516	0.103	2.594	0.560	

*p < 0.05

**p < 0.01

***p < 0.001

When admission variables and treatment variables were included in the multivariate logistic regression analysis, the significant independent variables were age (*p* < 0.05), GCS M ≤ 3 on admission (*p* < 0.05), multiple injuries (*p* < 0.05), and mechanical ventilation (*p* < 0.01). Variables that showed marginal significance were EML (*p* = 0.067), ethylism (*p* = 0.073), warfarin (*p* = 0.088), surgery before arrival (*p* = 0.053), and evacuated contusions (*p* = 0.055) (Table 5). Age was studied as a continuous variable so for every year in age there was a 0.94 odds ratio for favorable outcome, meaning the chance of favorable outcome decreased 6% with each increase of 1 year in age.

**Table 5:** Prediction model of admission and treatment variables for favorable outcome (multivariate logistic regression analysis with favorable outcome (GOSE 5–8) as dependent variable)

Variables	Regression coefficient	SE	Wald *X*^2^	Odds ratio	95% CI		*p*	
					Lower	Upper		
Intercept	5.918	2.309	6.571	371.609			0.010	*
Age	−0.064	0.031	4.240	0.938	0.882	0.997	0.039	*
Sex (female)	0.019	0.394	0.002	1.019	0.471	2.204	0.962	
GCS M ≤ 3 on admission	−1.727	0.768	5.061	0.178	0.039	0.801	0.024	*
Multiple injuries	− 1.077	0.466	5.342	0.340	0.137	0.849	0.021	*
Under the influence of drugs/alcohol at trauma	− 0.152	0.535	0.081	0.859	0.301	2.449	0.776	
Marshall Classification								
Diffuse injury I–IV	0.508	0.547	0.861	1.661	0.569	4.855	0.353	
EML	− 1.157	0.631	3.359	0.314	0.091	1.084	0.067	
CT dominating injury type								
Extracerebral hematoma	0.070	0.541	0.017	1.073	0.371	3.098	0.897	
Contusions	0.564	0.449	1.575	1.757	0.729	4.238	0.210	
Medical history of								
Brain injury/disease	− 0.685	0.486	1.987	0.504	0.194	1.307	0.159	
Traumatic brain injury	− 1.398	1.313	1.133	0.247	0.019	3.241	0.287	
Diabetes mellitus	− 0.684	0.499	1.878	0.505	0.190	1.342	0.171	
Hypertension/CVD	0.158	0.419	0.141	1.171	0.515	2.664	0.707	
Ethylism	− 0.961	0.536	3.217	0.383	0.134	1.093	0.073	
Antithrombotic drugs								
Antiplatelet	0.212	0.465	0.209	1.237	0.497	3.075	0.648	
Warfarin	− 0.968	0.568	2.904	0.380	0.125	1.156	0.088	
NOAK	− 0.349	0.859	0.165	0.706	0.131	3.797	0.685	
LMWH	− 0.960	1.085	0.783	0.383	0.046	3.210	0.376	
Surgery before arrival	1.480	0.765	3.746	4.395	0.981	19.681	0.053	
Craniotomy	− 0.122	1.211	0.010	0.885	0.082	9.498	0.920	
Evacuated extracerebral hematoma	1.020	1.099	0.862	2.773	0.322	23.898	0.353	
Evacuated contusions	− 1.614	0.842	3.676	0.199	0.038	1.037	0.055	
Decompressive hemicraniectomy	− 1.202	1.078	1.243	0.301	0.036	2.488	0.265	
Multiple surgeries	0.473	0.614	0.595	1.605	0.482	5.344	0.441	
ICP monitoring	0.489	0.433	1.276	1.630	0.698	3.806	0.259	
Mechanical ventilation	− 1.637	0.548	8.910	0.195	0.066	0.570	0.003	**

*p < 0.05

**p < 0.01

***p < 0.001

## Discussion

Forty-six percent of the elderly over 60 years of age had favorable outcome (GOSE 5–8), while 27% had unfavorable outcome (GOSE 2–4), and 27% died (GOSE 1) (Fig. 3), which indicates that NIC may be beneficial for the elderly. The rate of favorable outcome was virtually unchanged up to 75 years of age and then a slight decrease was seen with more advanced age. Unfavorable outcome did not increase after 75 years of age; it appears as the reason for the slight decrease in the proportion of favorable outcome above 75 years of age was higher mortality rather than an increased proportion of unfavorable outcome (Fig. 3). Those results are important to consider when to decide to offer NIC or not in an elderly TBI patient, taking also into consideration the general assumption that elderly are not afraid to die but to become dependent [41]. It should be emphasized, however, that these results cannot be extrapolated to the elderly population in general, since there was a selection of elderly patients judged to have a reasonable chance to achieve favorable outcome depending on, e.g., previous functional status, type of injury, level of consciousness, and co-morbidity. It is important to look at the characteristics of the elderly patients studied and try to identify prognostic factor in order to facilitate the selection of elderly TBI patients for NIC in the future.

The main cause of trauma in all elderly age groups was fall (Table 1), which is in accordance with our earlier findings [22, 28] as well as with the results of many other studies [8, 15, 17, 19, 23, 34, 36, 40]. Although there was a predominant injury mechanism, there was a notable significant difference between the age groups regarding several other characteristics (Table 1). The 60–74 years old were more often intoxicated at the time of trauma (26% vs 6%) and other injuries (25% vs 10%). They were also more likely to have contusions (35% vs 10%) and less likely to have ASDH (34% vs 76%). They had fewer cases of hypertension/CVD (48% vs 72%) and antithrombotic drugs (30% vs 62%, warfarin 8% vs 42%) and were more likely to have a history of ethylism (30% vs 10%). These findings highlight important differences between the 60–74-year-old group, and the 75–89-year-old group. The differences were also reflected in patient management with the older group having more craniotomies than the younger group (60% vs 38%). This may be explained by the fact that ASDH was more common among patients 75–89 years old and consistently it was also found that the reason for craniectomy was ASDH in 56% in the older age group compared to 31% in the younger group (Table 2).

Looking for prognostic predictors in the medical history, none of the following, such as previous brain injury/disease, previous traumatic brain injury, diabetes mellitus, and ethylism, had any significant impact on favorable outcome in the univariate analysis or the multivariate analyses, which was unexpected (Tables 3, 4, and 5). This of course does not exclude that those factors do not influence clinical outcome, but simply means that we were unable to show significant differences with our data. The reasons for that may be that some of those factors were present in too large proportions of the patients and others in too small proportions, and that a larger patient material is required to show significant differences in outcome. It is obvious that established prognostic factors from large patient materials of all ages cannot be disregarded in the decision-making process for which elderly TBI patients should be treated.

Antithrombotic drugs as a group had no negative impact on outcome in the univariate analysis. However, in a subgroup analysis, warfarin was a significant prognostic factor and antiplatelet therapy showed marginal significance (*p* = 0.053), but neither showed any significant independent contribution in the multivariate analysis (Table 4, Table 5). This finding is in contrast to the results of many earlier studies and needs to be discussed in particular. Karni et al. found a 50% mortality rate for traumatic head injury in elderly with anticoagulants [18]. Lavoie et al. showed that preinjury warfarin in elderly with closed head injury had more severe head injury and a higher likelihood of death [20]. Franko et al. showed that warfarin carries a six-fold increase in TBI-mortality and that mortality and occurrence of intracerebral hemorrhage increased with higher international normalized ratio (INR), especially INR over 4.0 where the mortality was found to be 50% and the risk of intracerebral hematoma (ICH) 75% [12]. Grandhi et al. found that warfarin and not antiplatelet medication influenced survival and need for neurosurgical intervention in the elderly [14]. Pieracci et al. found that the degree of anticoagulation rather than warfarin itself predicts adverse outcome in TBI in elderly patients [35]. Ohm et al. showed that elderly with intracranial hemorrhage and antiplatelet therapy had increased mortality [32]. Wong et al. found in their study that clopidogrel increased mortality but not warfarin and aspirin [45]. There are also contradicting studies. In 2017, Ganetsky et al. examined 939 patients who had ground-level falls and antiplatelet therapy or anticoagulants, and found a low incidence of clinically significant intracranial hemorrhage (< 5%) and no difference between anticoagulation and antiplatelet therapy [13]. One could speculate that possible reasons for why anticoagulants did not have any prognostic significance in our study could be: (1) In our referral area, patients on warfarin have frequent check-ups which reduces the risk for overtreatment with too high INR. (2) National guidelines require CT examination after mild head trauma when on anticoagulation and prompt reversal of warfarin in case of intracranial hemorrhages. (3) Standardized NIC which minimizes secondary insults may prevent worsening of intracranial hemorrhages. Altogether, however, it is reasonable to assume that anticoagulation therapy increases the risk for worsening of the head injury and may under some circumstances complicate the insertion of ICP devices and surgical treatment, although such therapy doses not make successful management impossible.

Considering other possible prognostic factors analyzed in the univariate analysis, diffuse injury I–IV had a OR > 1 and seems to be associated with favorable outcome (most likely due to the large number of diffuse injury II, the least serious class in that group). EML had an OR 0.299 indicating less chance of good outcome (Table 3). Both craniotomy and evacuated extracerebral hematoma had a negative influence on good outcome in the univariate analysis as well as mechanical ventilation (Table 3).

When analyzing potential prognostic factors, it is of utmost importance to identify factors with independent prognostic information. The multivariate analysis of prognostic admission factors for favorable outcome showed that high age and multiple injuries had a significant independent negative prognostic value and low GCS showed marginal significance (*p* = 0.052) (Table 4), which was as expected and in accordance with other studies of elderly patients [6, 29, 38, 44]. When both treatment factors and admission factors were included in the multivariate analysis of prognostic factors for favorable outcome, age, low GCS, and multiple injuries all had significant independent negative prognostic value. Surgery before arrival (evacuated ASDH at the referring hospital) showed positive prognostic value of marginal significance (*p* = 0.053). Evacuation of contusions and extracerebral hematoma, which were significant prognostic factors in the univariate analysis, did not show any significant independent influence on clinical outcome, although evacuation of contusions had marginal significant (*p* = 0.055). Mechanical ventilation on the other hand proved to have independent negative predictive value for favorable outcome (OR 0.195) (Table 5). The reasonable explanation for that may be that mechanical ventilation is not completely dependent on the severity of brain injury but also related to other factors not included in the statistical analysis, e.g., various infections including lung infections and other adverse events. Barnato et al. also found that elderly treated at the intensive care unit who survived mechanical ventilation had worse functional outcome [2]. It is likely that the negative impact of mechanical ventilation on outcome depends both on a more severe brain injury requiring mechanical ventilation, and on the development of systemic complications, with which the elderly are less able to cope.

There are some study limitations that needs to be considered. This is a single-center study and the results may have been influenced by the local management applied, and therefore the results may not be completely generalizable. Furthermore, as mentioned earlier, there was a selection bias since predominantly patients judged to have a reasonable chance for favorable outcome were accepted for NIC. Therefore, the results need to be interpreted with caution.

While these results may at first look discouraging but for this group of elderly TBI patients, a relatively large proportion achieved favorable outcome, when they were treated according to modern NIC principles and the treatment did not cause a large proportion of patients with severe disability or vegetative state. Similar results have also been reported by others [24, 29, 37, 47]. Further studies are required focusing on the NIC specifically in elderly TBI patients concerning, e.g., secondary insults, ICP management, and cerebral perfusion thresholds, to find out if these areas holds the key to improve outcome.

## Conclusion

This study shows that an appropriately selected group of elderly TBI patients receiving modern NIC have a fair chance of favorable outcome without large risks for severe deficits and vegetative state. Significant negative prognostic factors were high age, multiple injuries, low GCS M on admission, and the use of mechanical ventilation. The results underline that elderly with TBI should have access to NIC, when favorable outcome is as high as 47% for patients 60–74 years and around 30% for the patients between 75 and 84 years. Further research is needed about the selection of elderly patients and the optimal NIC management of elderly with TBI.

## Abbreviations

TBI: Traumatic brain injury
NIC: Neurointensive care
GOSE: Extended Glasgow Outcome Scale
GCS: Glasgow Coma Scale
GCS M: Glasgow Coma Scale motor response
ATLS: Advanced Trauma Life Support
CVD: Cardiovascular disease
ICP: Intracranial pressure
CT: Computed tomography
EVD: External ventricular drain
CPP: Cerebral perfusion pressure
SBP: Systolic blood pressure
CVP: Central venous pressure
CSF: Cerebrospinal fluid
ASDH: Acute subdural hematoma
EML: Evacuated mass lesions
INR: International normalized ratio
ICH: Intracerebral hematoma
OR: Odds ratio
EDH: Epidural hematoma
NOAC: Non-vitamin K antagonist oral anticoagulants
LMWH: Low molecular weight heparin

## References

[1] Andrews BT, Pitts LH (1991). Functional recovery after traumatic transtentorial herniation. Neurosurgery.

[2] Barnato AE, Albert SM, Angus DC, Lave JR, Degenholtz HB (2011). Disability among elderly survivors of mechanical ventilation. Am J Respir Crit Care Med.

[3] Becker DP, Miller JD, Ward JD, Greenberg RP, Young HF, Sakalas R (1977). The outcome from severe head injury with early diagnosis and intensive management. J Neurosurg.

[4] Bowers SA, Marshall LF (1980). Outcome in 200 consecutive cases of severe head injury treated in San Diego County: a prospective analysis. Neurosurgery.

[5] Cheng C, Chi NC, Williams E, Thompson HJ (2018) Examining age-related differences in functional domain impairment following traumatic brain injury. Int J Older People Nursing:e12208. 10.1111/opn.12208 10.1111/opn.12208 PMC6251737 30129175

[6] Chestnut RM, Ghajar J, Maas AIR, Marion DW, Servadei F, Teasdale G, Unterberg A, von Holst H, Walter BC (2000). Part 2: early indicators of prognosis in severe traumatic BRAIN injury. J Neurotrauma.

[7] Combes P, Fauvage B, Colonna M, Passagia JG, Chirossel JP, Jacquot C (1996). Severe head injuries: an outcome prediction and survival analysis. Intensive Care Med.

[8] Dams-O’Connor K, Cuthbert JP, Whyte J, Corrigan JD, Faul M, Harrison-Felix C (2013). Traumatic brain injury among older adults at level I and II trauma centers. J Neurotrauma.

[9] Eker C, Asgeirsson B, Grande PO, Schalen W, Nordstrom CH (1998). Improved outcome after severe head injury with a new therapy based on principles for brain volume regulation and preserved microcirculation. Crit Care Med.

[10] Elf K, Nilsson P, Enblad P (2002). Outcome after traumatic brain injury improved by an organized secondary insult program and standardized neurointensive care. Crit Care Med.

[11] Fischerström A, Nyholm L, Lewén A, Enblad P (2014). Acute neurosurgery for traumatic brain injury by general surgeons in Swedish county hospitals: a regional study. Acta Neurochir.

[12] Franko J, Kish KJ, O’Connell BG, Subramanian S, Yuschak JV (2006). Advanced age and preinjury warfarin anticoagulation increase the risk of mortality after head trauma. J Trauma.

[13] Ganetsky M, Lopez G, Coreanu T, Novack V, Horng S, Shapiro NI, Bauer KA (2017). Risk of intracranial hemorrhage in ground-level fall with antiplatelet or anticoagulant agents. Acad Emerg Med.

[14] Grandhi R, Harrison G, Voronovich Z, Bauer J, Chen SH, Nicholas D, Alarcon LH, Okonkwo DO (2015). Preinjury warfarin, but not antiplatelet medications, increases mortality in elderly traumatic brain injury patients. J Trauma Acute Care Surg.

[15] Hawley C, Sakr M, Scapinello S, Salvo J, Wrenn P (2017). Traumatic brain injuries in older adults-6 years of data for one UK trauma Centre: retrospective analysis of prospectively collected data. Emerg Med J.

[16] Hukkelhoven CW, Steyerberg EW, Rampen AJ, Farace E, Habbema JD, Marshall LF, Murray GD, Maas AI (2003). Patient age and outcome following severe traumatic brain injury: an analysis of 5600 patients. J Neurosurg.

[17] Iaccarino C, Carretta A, Nicolosi F, Morselli C (2018). Epidemiology of severe traumatic brain injury. J Neurosurg Sci.

[18] Karni A, Holtzman R, Bass T, Zorman G, Carter L, Rodriguez L, Bennett-Shipman VJ, Lottenberg L (2001). Traumatic head injury in the anticoagulated elderly patient: a lethal combination. Am Surg.

[19] Koskinen S, Alaranta H (2008). Traumatic brain injury in Finland 1991-2005: a nationwide register study of hospitalized and fatal TBI. Brain Inj.

[20] Lavoie A, Ratte S, Clas D, Demers J, Moore L, Martin M, Bergeron E (2004). Preinjury warfarin use among elderly patients with closed head injuries in a trauma center. J Trauma.

[21] LeBlanc J, de Guise E, Gosselin N, Feyz M (2006). Comparison of functional outcome following acute care in young, middle-aged and elderly patients with traumatic brain injury. Brain Inj.

[22] Lenell S, Nyholm L, Lewen A, Enblad P (2015). Updated periodic evaluation of standardized neurointensive care shows that it is possible to maintain a high level of favorable outcome even with increasing mean age. Acta Neurochir.

[23] Lennartsson C, Heimerson I (2012). Elderly people’s health: health in Sweden: the National Public Health Report 2012. Chapter 5. Scand J Public Health.

[24] Li LF, Lui WM, Wong HH, Yuen WK, Leung GK (2017). Outcome after operative intervention for traumatic brain injuries in the elderly. Asian J Neurosurg.

[25] Marshall LF, Marshall SB, Klauber MR, Clark MB, Eisenberg HM, Jane JA, Luerssen TG, Marmarou A, Foulkes MA (1991). A new classification of head injury based on computerized tomography. Spec Suppl.

[26] Marshall LF, Smith RW, Shapiro HM (1979). The outcome with aggressive treatment in severe head injuries. Part I: the significance of intracranial pressure monitoring. J Neurosurg.

[27] Marshall LF, Smith RW, Shapiro HM (1979). The outcome with aggressive treatment in severe head injuries. Part II: acute and chronic barbiturate administration in the management of head injury. J Neurosurg.

[28] Merzo A, Lenell S, Nyholm L, Enblad P, Lewen A (2016). Promising clinical outcome of elderly with TBI after modern neurointensive care. Acta Neurochir.

[29] Mosenthal AC, Lavery RF, Addis M, Kaul S, Ross S, Marburger R, Deitch EA, Livingston DH (2002). Isolated traumatic brain injury: age is an independent predictor of mortality and early outcome. J Trauma.

[30] Nordstrom CH, Sundbarg G, Messeter K, Schalen W (1989). Severe traumatic brain lesions in Sweden. Part 2: impact of aggressive neurosurgical intensive care. Brain Inj.

[31] Nyholm L, Howells T, Enblad P, Lewén A (2013). Introduction of the Uppsala Traumatic Brain Injury register for regular surveillance of patient characteristics and neurointensive care management including secondary insult quantification and clinical outcome. Ups J Med Sci.

[32] Ohm C, Mina A, Howells G, Bair H, Bendick P (2005). Effects of antiplatelet agents on outcomes for elderly patients with traumatic intracranial hemorrhage. J Trauma.

[33] Pedersen K, Fahlstedt M, Jacobsson A, Kleiven S, von Holst H (2015). A national survey of traumatic brain injuries admitted to hospitals in Sweden from 1987 to 2010. Neuroepidemiology.

[34] Peeters W, Majdan M, Brazinova A, Nieboer D, Maas AIR (2017). Changing epidemiological patterns in traumatic brain injury: a longitudinal hospital-based study in Belgium. Neuroepidemiology.

[35] Pieracci FM, Eachempati SR, Shou J, Hydo LJ, Barie PS (2007). Degree of anticoagulation, but not warfarin use itself, predicts adverse outcomes after traumatic brain injury in elderly trauma patients. J Trauma.

[36] Sjörgen H, Björnstig U (1991). Injuries among the elderly in the home environment:detailed analysis of mechanisms and consequences. J Aging Health.

[37] Stocchetti N, Paternò R, Citerio G, Beretta L, Colombo A (2012). Traumatic brain injury in an aging population. J Neurotrauma.

[38] Susman M, DiRusso SM, Sullivan T, Risucci D, Nealon P, Cuff S, Haider A, Benzil D (2002). Traumatic brain injury in the elderly: increased mortality and worse functional outcome at discharge despite lower injury severity. J Trauma.

[39] Teasdale GM, Pettigrew LE, Wilson JT, Murray G, Jennett B (1998). Analyzing outcome of treatment of severe head injury: a review and update on advancing the use of the Glasgow Outcome Scale. J Neurotrauma.

[40] Thomas KE, Stevens JA, Sarmiento K, Wald MM (2008). Fall-related traumatic brain injury deaths and hospitalizations among older adults--United States, 2005. J Saf Res.

[41] Unterhofer C, Ho WM, Wittlinger K, Thome C, Ortler M (2017). “I am not afraid of death”-a survey on preferences concerning neurosurgical interventions among patients over 75 years. Acta Neurochir.

[42] Wettervik TS, Lenell S, Nyholm L, Howells T, Lewen A, Enblad P (2018). Decompressive craniectomy in traumatic brain injury: usage and clinical outcome in a single centre. Acta Neurochir.

[43] Wilson JT, Pettigrew LE, Teasdale GM (1998). Structured interviews for the Glasgow Outcome Scale and the extended Glasgow Outcome Scale: guidelines for their use. J Neurotrauma.

[44] Vollmer DG, Torner JC, Jane JA, Sadovnic B, Charlebois D, Eisenberg HM, Foulkes MA, Marmarou A, Marshall LF (1991). Age and outcome following traumatic coma: why do older patients fare worse?. Spec Suppl.

[45] Wong DK, Lurie F, Wong LL (2008). The effects of clopidogrel on elderly traumatic brain injured patients. J Trauma.

[46] World Population Prospects (2017) The 2017 Revision, Volume II: Demographic Profiles. ST/ESA/SER.A/400. United Nations, Department of Economic and Social Affairs, Population Division

[47] Wutzler S, Lefering R, Wafaisade A, Maegele M, Lustenberger T, Walcher F, Marzi I, Laurer H, TraumaRegister DGU (2015). Aggressive operative treatment of isolated blunt traumatic brain injury in the elderly is associated with favourable outcome. Injury.

[48] Wärme PE, Bergström R, Persson L (1991). Neurosurgical intensive care improves outcome after severe head injury. Acta Neurochir.

